# Life Table Analysis for Immatures and Female Adults of the Predatory Beetle, *Delphastus catalinae*, Feeding on Whiteflies Under Three Constant Temperatures

**DOI:** 10.1673/031.008.0701

**Published:** 2008-01-28

**Authors:** Jesusa Crisostomo Legaspi, Benjamin C. Legaspi, Alvin M. Simmons, Mohamed Soumare

**Affiliations:** ^1^ USDA-ARS-CMAVE / Center for Biological Control, Florida A&M University, 6383 Mahan Drive, Tallahassee FL 32308; ^2^ FPSC, 2540 Shumard Oak Boulevard, Tallahassee, FL 32399; ^3^ U.S. Vegetable Laboratory, USDA-ARS, 2700 Savannah Highway, Charleston, SC 29414; ^4^ Center for Forest Ecosystems Assessment, Alabama A&M University, Normal, AL 35762

**Keywords:** Coccinellidae, developmental rate, survivorship, fecundity, *Bemisia tabaci*

## Abstract

Immature development and reproductive life history of *Delphastus catalinae* (Horn) (Coleoptera: Coccinellidae) feeding on *Bemisia tabaci* biotype B (Gennadius) (Homoptera: Aleyrodidae) (= *B. argentifolii* Bellows and Perring) immatures was studied at three constant temperatures: 22, 26 and 30 °C. Lower developmental threshold temperatures (*T0*) were estimated at 9 and 9.9 °C, for males and females, respectively. Female adults weighed slightly more than males (0.587 and 0.505 mg, respectively). As temperature increased from 22 to 30 °C, developmental time from eggs to eclosion of the adult declined from 24 to 15 days. Thermal units required for immature development was ∼300 degree-days. Percentage egg hatch declined at increasing temperatures, but no significant effect of time was found. The intrinsic rate of increase, *r*, increased from 0.048 to 0.082 and doubling time decreased from 14.44 to 8.45 days as temperature increased from 22 to 26 °C. Mean daily fecundity was modeled as a function of time and temperature to create a 3-dimensional surface. Overall, *Delphastus catalinae* was found to perform better at 22 and 26 °C while 30 °C was detrimental to immature development and adult reproduction.

## Introduction

The predatory beetle *Delphastus catalinae* (Horn) (Coleoptera: Coccinellidae) is an important whitefly predator. Before the genus was revised (Gordon 1994), this species was confused in the literature with *D. pusillus* (LeConte) (Hoelmer and Pickett 2003). It attacks whiteflies in citrus orchards and is used as a commercial biological control agent against several species of whiteflies, including biotype B of the sweetpotato whitefly, *Bemisia tabaci* (Gennadius) (= *B. argentifolii* Bellows and Perring) (Homoptera: Aleyrodidae) (Heinz et al. 1999; Liu and Stansly 1999), and the greenhouse whitefly, *Trialeurodes vaporariorum* (Lucas et al. 2004). Foraging behavior of *D. catalinae* may be affected by the presence of trichomes on host plant leaves, without adversely affecting prey consumption rate because the predator can walk on leaf trichomes while inserting its head between them to reach the leaf surface (Guershon and Gerling 1999). Compared with another coccinellid predator of whiteflies, *Nephaspis oculatus, D. catalinae* follows similar searching patterns, but moves at a higher rate when feeding on *B. tabaci* on hibiscus host plants (*Hibiscus rosa-sinensis*) (Liu and Stansly 1999).

Greenhouse and field experiments have shown that *D. catalinae* can suppress whitefly populations, with varying degrees of success. In field cage experiments on cotton, releases of *D. catalinae* resulted in >50% decreases in *B. tabaci* densities. However, no differences were detected in open field release experiments (Heinz et al. 1999). In greenhouse poinsettias (*Euphorbia pulcherrima*), releases of *D. catalinae* resulted in control against *B. tabaci* comparable to insecticidal treatments, but were about 5 times the cost (excluding indirect cost savings for environmental protection and worker safety) (Heinz and Parrella 1994a). Greater control, and perhaps reduced cost, may be achieved by using *D. catalinae* in conjunction with other compatible biological control agents. *D. catalinae* may be applied with parasitoids such as *Encarsia luteola* (Heinz and Parrella 1994a), *E. formosa* and *E. pergandiella*, to achieve enhanced control of *B. tabaci* (Heinz and Nelson 1996). Moreover, the use of *D. catalinae* as a biocontrol tool has been shown to be compatible with the use of sticky cards for whitefly sampling and population reductions (Simmons 2003; Simmons et al 2004). The coccinellid has shown a marked aversion to whitefly prey that were previously parasitized by *E. sophia* (=*E. transvena*) (Hoelmer et al. 1994). In this paper, we report the immature development and adult female life history of *D. catalinae* feeding on *B. tabaci* at three constant temperatures by following an entire generation from eggs stage until death of the adult females.

## Materials and Methods

### Temperature treatments

Methods used for studies on the effects of selected temperatures on the development of *D. catalinae* were similar to those described previously (Legaspi 2004; Legaspi and Legaspi 2005). *Delphastus catalinae* immatures and adult females were studied under three constant temperatures: 22, 26 and 36 °C. Constant temperature conditions were maintained inside ThermoForma Model 3740 growth chambers (ThermoForma, Marietta, OH) with a 14:10 (L:D) photoperiod and mean of 60% RH. Temperature and relative humidity inside each chamber were monitored by HOBO recorders (Onset Computer Corp., Bourne, MA, www.onsetcomp.com).

### *Delphastus catalinae* immatures

Fifty males and 50 females of *D. catalinae* adults were placed in a Petri dish (100 mm diameter) lined with filter paper. Food was provided by a piece of tomato leaf, *Solanum lycopersicum* L. =*Lycopersicon esculentum* Mill. (Solanales: Solanaceae) infested with *B. tabaci* nymphs. The tomato leaf served as an oviposition substrate. The leaf and filter paper were replaced daily. Water was provided by a soaked cotton ball placed in a 1-dram vial cap. Thirty newly-laid eggs of *D. catalinae* were placed individually in Petri dishes (100 mm diameter) for each temperature treatment. Body lengths (mm) of larvae were recorded at each immature stage. Larvae were supplied with sufficient food (∼200 *B. tabaci* nymphs daily) until adult eclosion. All Petri dishes were placed in sealed plastic containers with screen ventilation to prevent the immature and adult beetles from escaping. Time of egg hatch, duration of each immature lifestage and adult gender were recorded. Head coloration was used to distinguish males from females. The head of the males had an orange anterior whereas females had a black anterior section.

### *Delphastus catalinae* adult females

From the previous experiment, 10 adult females and 10 males (1-day old) were selected from each temperature treatment and maintained at the same temperature in which they developed, for a study of the reproductive life history of *D. catalinae*. Each adult female was placed with one adult male confined in a small Petri dish (100 mm diameter). Each caged pair of *D. catalinae* was provided an overabundance of food (∼200 *B. tabaci* nymphs on tomato leaves, replaced daily) and water until death of the female. Any dead males were replaced. Body weights of adults were recorded at weekly intervals with a BP221S balance (Sartorius Corp., Egdewood, NY, www.sartorius.com). Eggs were laid on both sides of the tomato leaf. Eggs were collected daily using a # 1 cork borer; leaf discs were then glued onto a filter paper (42.5 mm) and kept in a smaller dish (60 mm) and maintained in an environmental chamber at 26 °C until the eggs hatched. Times of oviposition and egg hatching, and numbers of eggs laid and hatched were recorded.

### Life table calculations

Reproductive parameters calculated using methods described previously (Southwood and Henderson 2000; Legaspi 2004) included: net reproductive rate (R0, mean number of female progeny produced by a single female during its mean lifetime, expressed in ♀/♀); gross reproductive rate (*GRR*, in ♀/♀); generation time (*T*, mean period between birth of the parents and that of the offspring, in days); intrinsic rate of increase (*r*, in ♀/♀/d); finite rate of increase (λ, in ♀/♀/d); and doubling time (*DT*, time for population to double, in days). Eggs were not reared to adulthood, so gender could not be determined. Assuming a 1:1 sex ratio, number of female eggs laid was estimated by dividing total eggs by two, as in previous studies (e.g. Wittmeyer and Coudron 2001).

### Statistical analyses

The effect of temperature on immature development rate (1/duration of life stage) was analyzed by linear regression. Separate analyses were performed on all immature stages from egg through pupae. Developmental threshold temperature was estimated as *T0* = -*a/b*. Degree days (*DD*) for development were calculated as *DD* = (*T* - *T0*) *D*, where *T* is the constant temperature used in the treatment and *D* is mean development time at that temperature (Greenberg et al. 2000). Adult body weights were analyzed using ANCOVA, with effects due to time, gender and temperature (time as covariate). Larval body lengths were analyzed as a 2-way ANOVA with effects due to instar stage and gender using Systat 11 (Systat Software, Inc., Point Richmond, California, www.systat.com)

For adults, one-way ANOVA was performed to analyze the effects of temperature on total number of eggs, age at first oviposition, percentage of eggs hatched, and body weight. Percentage data were converted using arcsine transformation, but are presented as untransformed means (Sokal and Rohlf 1995). Using nonlinear regression, a 3-dimensional surface was estimated to describe the effects of temperature and age on mean numbers of eggs laid daily. Mean numbers of eggs laid daily was fitted to the model: *eggs* = (*p* + *qT*) *d* exp (-*wTd*); where *T* is temperature (°C) and *d* is time (days) (Enkegaard 1993). The parameters *p* and *q* describe how quickly maximal oviposition is reached as a function of temperature; and *w* how quickly it returns to zero (Drost et al. 1988, Greenberg et al. 2000).

## Results and Discussion

### *Delphastus catalinae* immatures

#### Development rates

**Figure 1.**
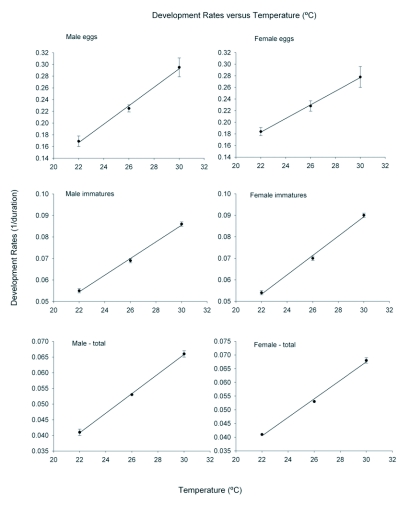
Developmental rate (1/development time) for immature *D. catalinae* as a function of temperature. Development rate (*Y*) was fitted to the equation *Y* = *a* + *bT*, where *T* is temperature (°C), *a* and *b* are constants. Development threshold was estimated as *T0* = (-*a*/*b*).

Immature developmental rates of *D. catalinae* significantly increased from 22–30°C (Table 1; Figure 1). Using equations for total development (egg to adult eclosion ), *T0* was 9 °C for immature males and 9.9 °C for females. In comparison, lower and upper threshold temperatures for survival over 24 hour durations were estimated at 0 and 40 °C for both pupae and adults (Simmons and Legaspi 2004). Thermal units required for immature development varied by gender and ranged from about 293 to 323 degree-days (Table 2).

#### Larval length and adult mass

Although larval lengths predictably increased with instar stage (*F* = 1171.04; df = 3, 276; *P* < 0.001; R^2^ = 0.93), there was no temperature effect (*F* = 0.157; df = 2, 276; *P* = 0.855; R^2^ = 0.93) (instar X temperature *F* = 3.58; df = 6, 276; *P* = 0.002; R^2^ = 0.93). Larval lengths (mm, mean ± SE) were 0.686 ± 0.009; 1.147 ± 0.018; 1.7126 ± 0.017; and 2.3347 ± 0.034 for 1^st^ to 4 instars, respectively. The ANCOVA model of adult weights was highly significant (*F* = 2.91; df = 254, 285; *P* < 0.001; R^2^ = 0.72) (Table 3). The significant effect was due to sex (*F* = 15.08; df = 1, 538; *P* = 0.000116; R^2^ = 0.03) with females weighing more than males (9: 0.587 ± 0.0084 SE; ♂: 0.505 ± 0.0008 SE). Adult weights were higher at 22 than 26 °C (Table 3), but did not change with time (Regression: *F* = 2.94; df = 1, 538; *P* = 0.087; R^2^ = 0.14).

**Table 1.**
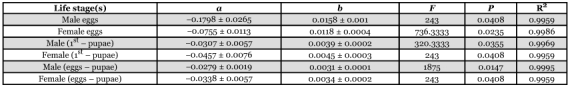
Parameter estimates for effects of temperature on development rate of *D. catalinae* immature lifestages using *Y* = *a* + *bx*; where *Y* is development rate, *x* is temperature (°C); *a* and *b* are constants (± SE).

**Table 2.**
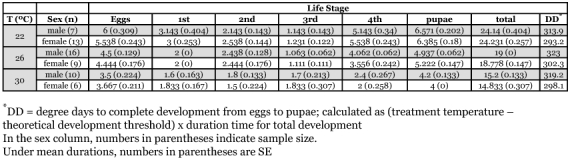
Life stage durations of *D. catalinae* at different temperatures.

#### Duration of immature lifestage

The total duration of the immature stages from egg to adult eclosion declined significantly from about 24 to 15 days as temperature increased from 22 to 30 °C (Table 2) (2-way ANOVA: *F* = 842.8; df = 2, 55; *P* < 0.001). However, development time did not differ between males and female immatures (*F* = 0.903, df = 1, 55; *P* = 0.346), nor was there a significant interaction (*F* = 0.549; df = 2, 55; *P* = 0.581). Measured immature development times are in agreement with a previous report of 21.0 days at 28 ± 3 °C (Hoelmer et al. 1993). Sex ratios determined upon eclosion to adulthood revealed 46% females (28 ♀: 32 ♂), thus supporting the prior decision to divide the number of eggs laid by 2 to estimate numbers of female eggs.

**Figure 2.**
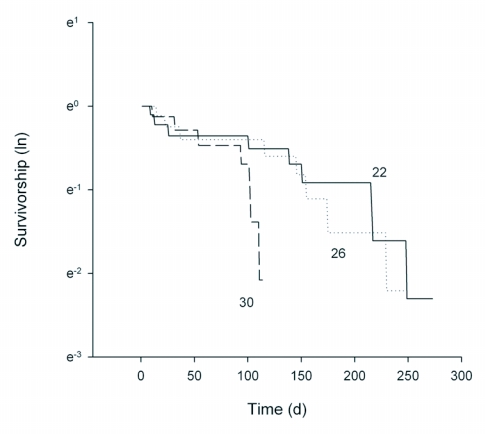
Survivorship curves of adult *D. catalinae* females (n = 10) when maintained under each of three constant temperature regimes.

### *Delphastus catalinae* adults

#### Oviposition measurements

Eggs were reared at the same temperatures to test for the effects of temperature regimen of the adult female on egg viability. Percentage of *D. catalinae* egg hatch was analyzed as a multiple regression wherein egg hatch was a function of both time and temperature. The regression was given as *Y* = 98.25 + 0.029*time* - 1.4*temp* (SE: 8.0, 0.01, and 0.30, *t*: 12.27^**^, 1.8 ns, 4.7^**^) (model *F* = 17.45; df = 2, 1435; *P* < 0.001; R^2^ = 0.024). Therefore, percentage egg hatch declined at increasing temperatures, but no significant effect of time was found.

**Table 3.**
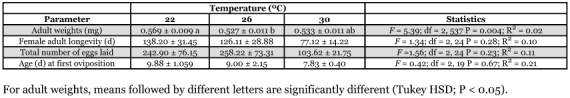
Effects of temperature on various oviposition parameters in *D. catalinae* (± SE).

#### Life table calculations

**Figure 3.**
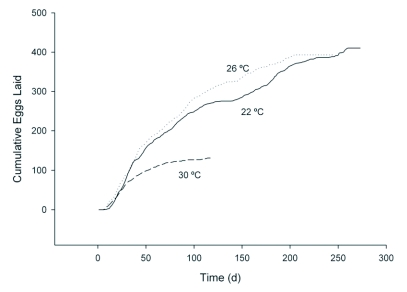
Mean cumulative eggs laid per female *D. catalinae* at 22, 26 and 30 °C.

The survivorship curve for female *D. catalinae* adults shows similar trends at 22 and 26 °C (Figure 2), with a shorter curve at 30 °C. However, there was much variation among individuals and the total numbers of eggs laid and adult female longevities were not significantly affected by temperature (Table 3). Cumulative numbers of eggs laid per female again showed similar trends at 22 and 26 °C, with a shorter curve at 30 °C (Figure 3). Life table parameters (Table 4) indicate that the intrinsic rate of increase *r*, increased from 0.048 to 0.082 and doubling time decreased from 14.44 to 8.45 days as the temperature increased from 22 to 30 °C. Mean adult female longevity ranged from 138 to 77 days, but the values were not statistically significant (Table 3). In comparison, Heinz and Parrella (1994b) measured adult female longevity of *D. catalinae* as 85.2 days at 27.6 °C (range: 25.8–30.1 °C), while Simmons and Legaspi (2004) noted that adults survived up 174 days at 25 °C and 18 days at 35 °C. Hoelmer et al. (1993) reported that longevity of adults was 60.5 days for females and 44.8 days for male adults at 28 °C (± 3). Liu (2005) studied the life history of *D. catalinae* feeding on *B. tabaci* on collards in the laboratory (∼26 °C). Developmental time for eggs, 1^st^ through 4^th^ instar larvae, and pupae were 4.0, 1.9, 1.1, 1.4, 5.2, and 5.3 d, respectively. Adult longevity was 122.6 days for females, 170.5 days for males and 146.6 days for both sexes combined. Estimates of reproductive parameters were: *R0* = 276.8; *GRR* = 325.1; *T* = 35.6; *DT* = 4.8 d; *r* = 0.158; λ = 1.171. In comparison, corresponding values at 26 °C in this study were *R0* = 129.437; *GRR* = 196.563; *T* = 80.76; *DT* = 11.51 d; *r* = 0.0602; λ = 1.062 (Table 4).

**Table 4.**
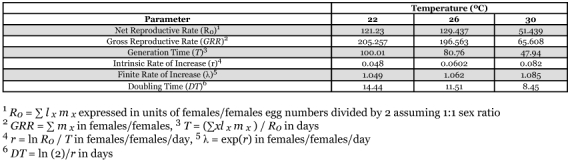
Life table parameters for adult *Delphastus catalinae* maintained under one of three constant temperature regimes.

**Figure 4.**
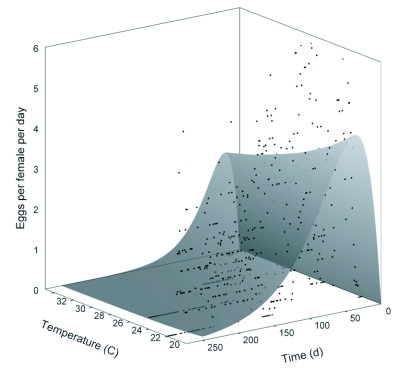
Enkegaard (1993) surface equation overlaid on mean numbers of *D. catalinae* eggs deposited as a function of age and temperature. The equation fitted was *eggs* = (0.319283 + 0.000015*T*) *d* exp (-0.001355*Td*), where *eggs* is mean eggs laid, *T* is temperature (°C) and *d* is age in days.

The Enkegaard equation models fecundity as a function of age and temperature (Drost et al. 1988). For *D. catalinae*, the equation was estimated as: *eggs* = (0.319283 + 0.000015*T*) *d* exp (-0.001355*Td*) (SE-values: 0.071394, 0.002790, 0.000043, for *p*, *q*, and *w*, respectively; *d* and *T* are age (days) and temperature (°C), respectively; *F* = 453.3; df = 3, 633; *P* < 0.01; R^2^ = 0.68). As shown in Figure 4 the surface indicates steep increases in oviposition rates early in the life of the female, with a gradual decline for the duration of its lifetime. The equation has been applied to whiteflies: *Bemisia* spp. (Drost et al. 1988; Greenberg et al. 2000) and *T. vaporariorum*. (Greenberg et al. 2000); thrips, *Frankliniella occidentalis* (Wang and Shipp 2001), a sugarcane borer parasite, *Allorhogas pyralophagus* (Harbison et al. 2001) and *Podisus maculiventris* (Legaspi and Legaspi 2005).

Liu (2005) concluded that the life history parameters equaled or exceeded those for *B. tabaci* under most greenhouse conditions, suggesting *D. catalinae* should succeed as a control agent. However, reproductive parameters reported in Liu (2005) are higher than those in the present study (Table 4) despite use of the same host insect, *B. tabaci*. Possibly significant was the use of collards as the host plant, as opposed to tomato in the present study. Furthermore, direct comparisons across different studies is complicated by the fact that the various authors cited used poinsettia, tomato, hibiscus, collards and possibly other plants. Lower reproductive parameters need not eliminate *D. catalinae* as a prospective control agent against *B. tabaci* because several studies have demonstrated its voracity as a predator of whiteflies (e.g., Heinz et al. 1999), especially whitefly eggs (e.g., Hoelmer et al. 1993; Legaspi et al. 2006).
